# Investigation of Ion Channel Activities of Gramicidin A in the Presence of Ionic Liquids Using Model Cell Membranes

**DOI:** 10.1038/srep11935

**Published:** 2015-07-20

**Authors:** Hyunil Ryu, Hwankyu Lee, Seigo Iwata, Sangbaek Choi, Moon Ki Kim, Young-Rok Kim, Shinsaku Maruta, Sun Min Kim, Tae-Joon Jeon

**Affiliations:** 1 Department of Biological Engineering, Inha University, Incheon 402-751, South Korea; 2 Biohybrid Systems Research Center (BSRC), Inha University, Incheon 402-751, South Korea; 3 Department of Chemical Engineering, Dankook University, Yongin-si, Gyeonggi-do 448-701, South Korea; 4 Division of Bioinformatics, Graduate School of Engineering, Soka University, Tokyo 192-8577, Japan; 5 School of Mechanical Engineering and SKKU Advanced Institute of Nanotechnology (SAINT), Sungkyunkwan University, Suwon, 440-746, Korea; 6 Institute of Life Sciences and Resources, and Department of Food Science and Biotechnology, Kyung Hee University, Yongin 446-701, South Korea; 7 Department of Mechanical Engineering, Inha University, Incheon 402-751, South Korea

## Abstract

Ionic liquids (ILs) are considered to be green solvents because of their non-volatility. Although ILs are relatively safe in the atmospheric environment, they may be toxic in other environments. Our previous research showed that the cytotoxicity of ILs to biological organisms is attributable to interference with cell membranes by IL insertion. However, the effects of ILs on ion channels, which play important roles in cell homeostasis, have not been comprehensively studied to date. In this work, we studied the interactions between ILs and lipid bilayer membranes with gramicidin A ion channels. We used two methods, namely electrical and fluorescence measurements of ions that permeate the membrane. The lifetimes of channels were increased by all the ILs tested in this work via stabilizing the compressed structure of the lipid bilayer and the rate of ion flux through gA channels was decreased by changing the membrane surface charge. The former effect, which increased the rate of ion flux, was dominant at high salt concentrations, whereas the latter, which decreased the rate of ion flux, was dominant at low salt concentrations. The effects of ILs increased with increasing concentration and alkyl chain length. The experimental results were further studied using molecular dynamics simulations.

Ionic liquids (ILs) are salts that are in the liquid state below 100 °C; they consist of organic cations and inorganic anions, and have wide liquid-temperature ranges. ILs are excellent solvents; they can dissolve inorganic and organometallic compounds and are capable of dissolving organic chemicals, including plastics, DNA, and crude oil^1^. Because of these properties, ILs have attracted attention as alternatives to common volatile organic solvents^2^, and because of their wide electrochemical window ranges, ILs have been developed as battery electrolytes^3^. The use of ILs as catalysts in organic reactions has been widely studied^4^. ILs have low vapor pressures and high thermal stabilities, and do not cause air pollution, therefore they are considered to be “ecofriendly solvents”^5^,^6^ in the chemical and pharmaceutical industries. Because ILs can be synthesized by combining cations and anions, they are also referred to as “designer solvents”^7^. Given the unique properties of ILs, they can replace conventional solvents used in the fields of biopolymers^8^, biosensors^9^, and cellulose processing^10^.

However, it is premature to define ILs as “green solvents,” as their toxicities and ecotoxicities have not been comprehensively investigated. Although ILs are considered the be relatively safe in the atmosphere, because of their high solubility, it is difficult to say whether ILs are safe in aquatic environments^11^. It has been reported that ILs are more aquatically toxic than conventional solvents are^12^,^13^. Moreover, ILs can be fatal to organisms, because they cannot be biodegraded and accumulate within the body^14^,^15^. ILs are therefore not perfect green solvents, and their toxicity mechanisms need to be thoroughly studied. However, only the toxicities of ILs to organisms have been studied^11^ and IL effects on ion channels have not been studied to date. ILs have many potential applications, therefore their toxicity mechanisms should be studied to enable safer IL design and synthesis.

In an investigation of the effects of ILs on cell membranes, Jeong *et al*. observed the morphological influences of ILs on *Shwanella oneidensis MR-1*^16^. To verify that ILs rupture membranes, they constructed a model membrane and mimicked interactions between the membrane and ILs. Their experimental results supported the theory that ILs are incorporated into the membrane. Moreover, the higher the concentration and the longer the hydrocarbon chain of the IL was, the more impact the IL had on the membrane. Their results agree well with previous results on cell toxicity^17^, and their investigation of the effects of ILs on membranes contributed to elucidating the cellular mechanisms of ILs. Conventional studies have only investigated the effects of ILs on membranes, but ion channel functions are influenced by the membrane, therefore ILs can affect ion channels as well^18^. To investigate the effects of ILs on the environment thoroughly, the influence of ILs on membranes and the interactions between membrane proteins, cell membranes, and ILs should be studied.

In this study, we constructed a model system that could be used to study the influence of ILs on lipid bilayer membranes and ion channels. We chose gramicidin A (gA) as our model ion channel, which is well suited to investigating membrane changes^19^, to identify the interactions of lipid bilayer membrane with ionic liquids in the presence of ion channels. gA has two subunits on each leaflet of a bilayer membrane and the subunits are associated to form a dimer. When a dimer is formed, gA selectively allows permeation of a monovalent cation through the pore. The rate of ion flux through gA channels and the lifetime of the gA dimer are affected by the surface charge and compressed structure of lipid bilayer near the gA dimer, respectively^19^,^20^. We therefore postulated that the ion channel activities (i.e. life time of gA dimer, conductance of gA, channel appearance rate) will be changed, because ILs affect the electrostatic and mechanical properties of the membrane.

To identify the effects of ILs on gA ion channels, we conducted electrical and fluorescence experiments. For the electrical measurement, we reconstituted a free-standing lipid bilayer with gA and recorded the lifetimes of gA ion channels and conductance changes in the presence of different concentrations and types of ILs. As a result, we were able to measure changes in rate of ion flux. To obtain experimental results at low salt concentrations, we conducted fluorescence experiments using liposomes containing a fluorophore. In both experiments, the longer the hydrocarbon carbon chain and the higher the concentration of ILs were, the more effectively the rate of ion flux through gA channels changed. To explain our experimental results further, molecular dynamics (MD) simulations were performed. The presence of ILs stabilizes the gA dimer and allows the permeation of more ions by increasing channel openning time, but ILs also change the membrane surface charge, which decreases rate of cation flux through each gA ion channel. Although both effects occur at the same time, one dominates over the other depending on ionic strength of solvents. Our findings on ion channel activities in the presence of ILs will contribute to designing more biocompatible ILs. For instance, our research will help to determine the appropriate alkyl chain length and functional head groups of ILs to discover more biocompatible ionic liquids^21^,^22^.

## Results

### Electrical measurements of ion flux through gA channels

A gA monomer is a single-stranded, right-handed β-helical polypeptide with 15 amino acids. In a bilayer, each isolated monomer diffuses laterally within each monolayer. When two gA monomers on a membrane are brought into contact, they form formyl end-bonds via six head-to-head hydrogen bonds^23^, with bond energies of 70–80 kJ/mol^24^,^25^. However, neighboring phospholipids are compressed on dimerization, because the length of the gA dimer is less than the membrane thickness. The structural changes in the bilayer have energy costs^26^, therefore gA dimers dissociate. Here, thermal equilibrium is established between the gA monomer and dimer^24^,^25^,^27^:


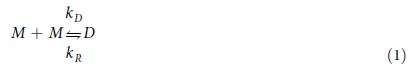


where M and D represent the gA monomer and dimer, respectively, and *k*_*D*_ and *k*_*R*_ represent the dissociation and association rate constants, respectively. The dissociation constant (*k*_*D*_) is the reciprocal of the channel lifetime (τ) which can be expressed as a function of the bilayer deformation energy. The bilayer deformation energy (*ΔG*_*def*_) is influenced by the hydrophobic mismatch between the channel length (*l*) and the bilayer thickness (*d*_*0*_), intrinsic curvature (*c*_*0*_), and elastic moduli (*Ka* and *Kc*). A short channel length for gA dimers will therefore create a positive mismatch with the bilayer thickness, affecting the channel lifetime (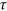). When 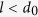, the more negative *c*_*0*_and the larger *d*_*0*,_*Ka* and *Kc* the higher *ΔG*_*def*_ becomes^19^.

**Figure 1:**
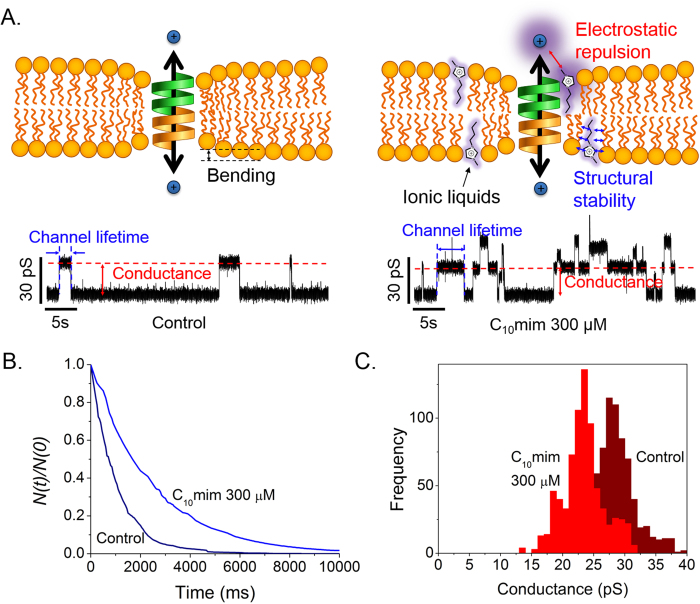
Lifetimes of gA dimer. (**A**) Effect of IL on gA channel reconstituted with DPhPC/*n*-decane lipid bilayer. Each current trace represents events prior to and after addition of 300 μM [C_10_ mim]Cl (applied voltage 200 mV). (**B**) Lifetime distribution of gA dimer depending on presence of IL. *N*(*t*) is the number of channels with lifetimes longer than time *t*. Blue line: C_10_ mim 300 μM; navy line: control. (**C**) Conductance transition amplitude histograms depending on presence of IL. Red: C_10_mim 300 μM; wine: control.

As described previously, ILs are incorporated into the lipid bilayer^16^, and therefore affect the deformation free energy of the bilayer membrane, thereby altering the lifetimes of gA dimers. We used an electrical assay to measure lifetime changes of gA ion channels. Typically, gA channels have a conductance of ~14 pS in 1 M NaCl; the ion channel conductance represents the amount of ions crossing the channel per unit time for a given applied voltage, and the duration of the conductance signal represents the gA dimer lifetime. We investigated the influence of ILs on the gA dimer lifetime by measuring the signal duration when ILs at various concentrations and with different alkyl-chain lengths were added. The membrane surface charge also changes when an IL is incorporated into the membrane, because of positive charges on the IL, creating an electrostatic barrier on the membrane surface (Fig. 1A). As membrane surface charge affects ion pearmeability of gA^20^,^28^, we measured the conductance of gA channels to determine the effects of surface charge to gA channels.

**Figure 2:**
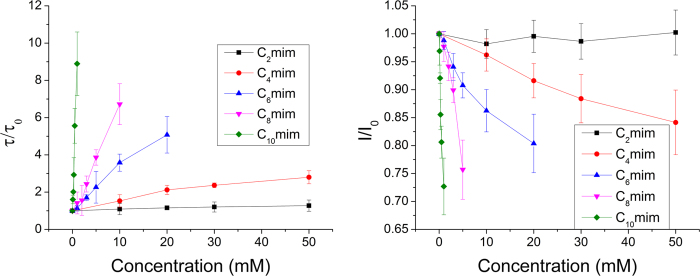
(**A**) Graph showing lifetime of gA dimer depending on types and concentrations of ILs (τ: lifetime of gA dimer with ILs, τ_0_: lifetime of gA without ILs). (**B**) Graph showing conductance of membrane depending on types and concentrations of ILs. I: current of gA with ILs, I_0_: current of gA without IL. 0, 0.1, 0.2, 0.3, 0.5, and 1 mM C_10_ mim; 0, 1, 2, 3, 5, and 10 mM C_8_ mim; 0, 1, 3, 5, 10, and 20 mM C_6_ mim; 0, 10, 20, 30, and 50 mM C_4_mim; and 0, 10, 20, 30, and 50 mM C_2_ mim were used. Each point represents more 700 data points form experiments conducted over three times.

Figure 1A shows a schematic illustration of the membrane and electrical measurement data in the presence/absence of ILs. Our previous study showed that the amount of ILs incorporated in a lipid bilayer depends on the length of the alkyl chain, therefore the chain length affects the lifetimes and ion permeability of gAs^16^. To show the significant effects of ILs with long alkyl chains, we performed lifetime and conductance measurements in the presence of C_10_ mim, which has 10 carbons in the alkyl tail. As shown in Fig. 1A, ILs incorporated in the bilayer decrease the deformation energy, increasing the lifetimes of the gA dimers. The data in Fig. 1B,C show that on addition of 300 μM C_10_ mim, the gA dimer lifetime and channel formation rate increased^19^ and the channel conductance decreased, as we hypothesized. In this work, however, we did not included data showing the influence of ILs on the channel appearance rate, the channel apperance rate cannot be precisely controlled due to the continuous association and dissociation of gA dimers, which is also caused by movement of gA from the solvent annulus to the bilayer and vice versa^29^. We therefore analyzed the lifetimes and conductances of the gA channels to clarify the channel behavior in the presence of various ILs. To demonstrate the effects of alkyl-chain length and IL concentration, we tested five different ILs (C_2_ mim, C_4_ mim, C_6_ mim, C_8_ mim, and C_10_ mim) with different alkyl-chain lengths. The results in Fig. 2 show thatwhen the same amounts of ILs were added to the solution, ILs with longer alkyl chains had greater effects on the gA activities; for example, the lifetime of the gA channel in 1 mM C_10_ mim was more than ~10 times longer than that in 1 mM C_8_ mim, although C_10_ mim has only two more alkyl chains. This is because of the increased hydrophobicity resulting from the increased length of the alkyl chain, which increases the incorporation rate into the bilayer, as well as the influences of ILs on *c*_*0*,_ *d*_*0*,_ *Ka* and *Kc* of the bilayer. Our results agree well with those of previous studies by Ingolfsson and Anderson on the influence of alcohol alkyl-chain length on gA^30^, in which alcohols with relatively short alkyl chains (C < 10) influenced gA activities as the length of the alkyl chain increased. Moreover, the lifetime of the gA dimer increases as the IL concentration increases (Fig. 2A). These results shown are attributed to stabilization of gA dimers by the decrease in bilayer deformation energy when ILs, which are shorter than lipids, are incorporated into the membrane^19^. Therefore, ILs tested in the work stabilized the compressed structure of lipid bilayer near gA dimers and consequently prolonged the lifetime of gA dimers. In contrast, the gA conductance decreased with increasing IL concentration, as shown in Fig. 2B. Because gA dimers form cation-selective ion channels^31^, the positively charged imidazole rings of ILs interfere with cation permeation through gA channels. Bruno *et al*. investigated conductance changes in the presence of cationic substances on membranes, and reported that the gA conductance decreased with increasing cation concentration^32^. Our results show that the decreases in gA channel conductance are mainly caused by changes in the charges on the membrane surface. Moreover, the conductance decreased as the length of the IL increased, because larger amounts of ILs with long alkyl chains were incorporated in the membrane.

### Fluorescence measurements of ion flux using liposomes

**Figure 3:**
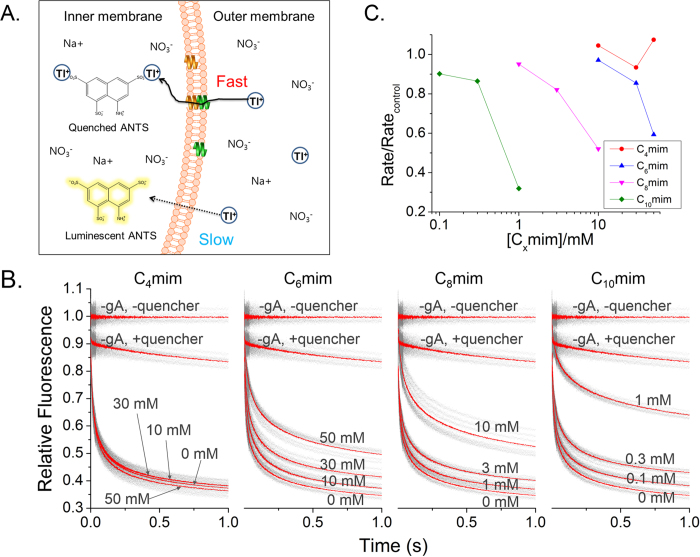
(**A**) LUVs used in experiments with fluorophore (ANTS), which can be quenched by Tl^+^ ions; gA is incorporated into the membrane. Tl^+^ ion is a monovalent cation, therefore it can pass through the pore of gA dimers; permeation of Tl^+^ ions through the membrane is slower. (**B**) Graph of timeline showing fluorescence intensity after inserting Tl^+^, a quenching molecule, into ANTS-encapsulated LUVs. Each experiment was conducted at pH 7, n > 6. 4 different ILs were used and each graph represents a different IL. The results for C_2_ mim are omitted as no change was observed. (**C**) Quenching rate comparison for each IL at 2 ms after reaction with Tl^+^ ion.

We used liposomes containing fluorophores to further study ion flux at low ionic strengths, because electrical measurements need to be done at high salt concentrations to discriminate the gA conductance signals. ANTS was encapsulated in liposomes and Tl^+^, which quenches ANTS, was placed outside the liposomes, as shown in Fig. 3A. The ANTS intensity, based on ANTS quenching fluorescence, changes with changes in rate of Tl^+^ ion influxinto vesicle. As shown in Fig. 3B, we tested influx of Tl^+^ ion into vesicle in the presence of four different ILs (C_4_ mim, C_6_ mim, C_8_ mim, and C_10_ mim). The ion permeability through the membrane without gA channels was also measured as a negative control, because a very small amount of ions may diffuse through the lipid bilayer^33^.

Figure 3B shows the normalized fluorescence intensities with various types and concentrations of ILs. The rate of ion influx, measured using fluorescence, decreased as the IL concentration increased. Data for ILs at concentrations higher than 50 mM could not be measured, because the ILs used are also fluorescent, creating background signals higher than the ANTS signal. In contrast to the results obtained using electrical measurements, the rate of ion flux through a membrane decreased as the IL alkyl-chain length and concentration increased. The effects of ILs on the rate of ion flux were greater when ILs with longer alkyl chains were used. Even small amounts of ILs with longer alkyl chains interfere with ion permeation through gA channel. Two reasons can be considered (Fig. 1A). The incorporation of ILs into membranes alleviates the deformation energy from membrane curvature attributed to short gA channels, resulting in greater ion permeability because of the longer lifetime and more frequent channel formation. However, the imidazole rings of ILs make the membrane surface positive, and this interferes with cation permeation through a gA channel. As shown in Fig. 3B, the latter effect is greater. Positive charges on the membrane surface significantly decrease the rate of ion flux due to cation depletion near gA channels^32^,^34^. To compare the effects of different ILs, we analyzed the ion permeability rate as a function of IL concentration at a fixed time, i.e., 2 ms, after mixing liposomes with Tl^+^. As shown in Fig. 3C, the surface charges on the membrane influenced ion permeation. ILs with longer alkyl chains are more efficiently incorporated into the membrane, therefore ion permeation was greatly reduced in the presence of very small amounts of C_10_ mim, whereas other ILs present at concentrations less than 1 mM did not significantly affect ion permeation. Almost no changes were observed when C_2_ mim and C_4_ mim which have less than four alkyl groups, were present in solutions up to concentrations of 50 mM. The ion permeability of the membrane can therefore be significantly influenced by positive charges on the membrane in solutions of low ionic strength, although the lifetimes of and channel formation by gA dimers should increase as a result of the decreased deformation energy. MD simulations were performed to analyze our experimental results comprehensively.

### Molecular dynamics simulations: electrical repulsion induced by ionic liquids

To understand the effects of ILs on the electrostatic interactions between lipid bilayers and cations, MD simulations of lipid bilayers containing C_10_ mim were performed at NaCl concentrations of 0.15 M and 1 M. Details of the simulated systems are shown in Table 1; in the table, “DOPC” and “C10 m”, respectively, denote the pure dioleoylglycerophosphocholine (DOPC) bilayer and the bilayer consisting of DOPC and C_10_ mim (molar ratio 7:1), and the molar concentrations of NaCl are denoted by “0.15” and “1”. Note that for the DOPC/C_10_ mim bilayer, more Cl^−^ ions are added as counterions to achieve electroneutrality.

**Figure 4:**
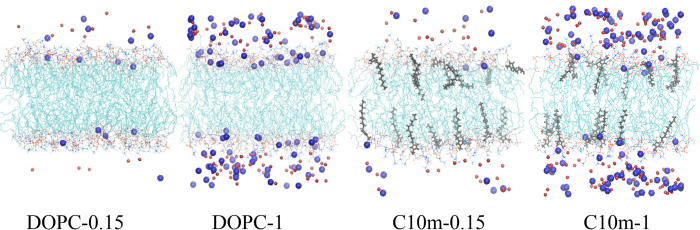
Snapshots at the end (40 ns) of simulations of pure DOPC bilayers and the DOPC/C_10_ mim bilayers. Gray thick lines represent C_10_ mim molecules, while blue, red, and light-blue thin lines represent the choline, phosphate, and tail groups of DOPC, respectively. Na^+^ and Cl^−^ ions are respectively colored in blue and red, and Na^+^ are highlighted in the larger dots. For clarity, water molecules are omitted. The images were created with Visual Molecular Dynamics 1.9.2 (http://www.ks.uiuc.edu/Research/vmd)^52^.

**Figure 5:**
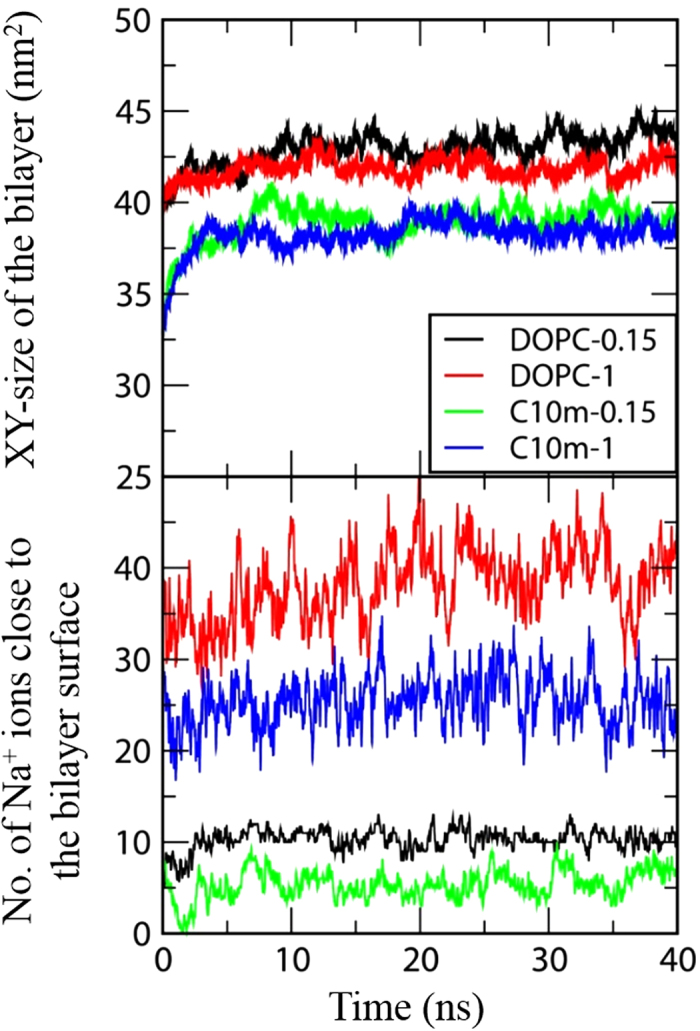
The bilayer size in XY dimension (top) and the number of Na^+^ ions close to the bilayer surface (bottom) as functions of time.

Figure 4 shows the final snapshots of the simulations. For the pure DOPC membranes, Na^+^ and Cl^−^ ions, respectively, bind to anionic phosphates and cationic cholines, as a result of electrostatic interactions; fewer Na^+^ ions interact with the bilayer surface of the DOPC/C_10_ mim membrane, indicating the effect of C_10_ mim on the interactions between ions and the bilayer. Figure 5 shows the *XY*-plane areas (which equal the bilayer surface areas) and the number of Na^+^ ions close to the membrane surface as functions of time. If the distance between Na^+^ and the phosphate atoms of DOPC is less than 0.8 nm, the corresponding Na^+^ ions are considered to be close to the membrane surface. In Fig. 5, both these values reach steady states within 20 ns, indicating that the membranes are equilibrated within the simulated timescale. The average numbers of Na^+^ ions around the bilayer are 10.5 (±0.1), 39.8 (±0.6), 5.7 (±0.4), and 26.5 (±0.4), respectively, for DOPC-0.15, DOPC-1, C10 m-0.15, and C10 m-1, showing that ILs reduce the extent of binding of Na^+^ in the bilayer, presumably because of the repulsive force between cationic C_10_ mim and Na^+^ ions. In particular, the extent of Na^+^ binding in the bilayer decreases in the bilayer with ILs at both 0.15 M and 1 M NaCl, but the effects differ. The levels of Na^+^ binding in the bilayer are reduced by 46% and 33%, respectively, at NaCl concentrations of 0.15 M and 1 M, indicating that the inserted C_10_ mim molecules induce less electrical repulsion at the higher NaCl concentration, apparently because of the weaker C_10_ mim–Na electrostatic force induced by the increased dielectric constant.

**Figure 6:**
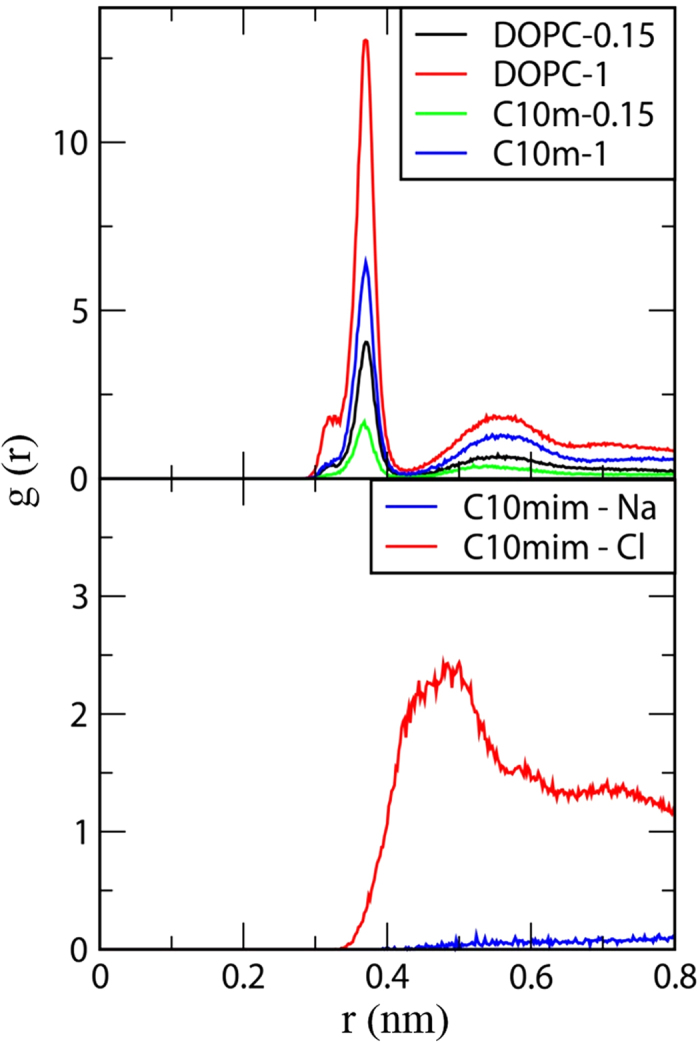
Radial distribution functions (RDF) for Na^+^ ions with respect to the phosphorus atoms of DOPC (top), and those for Na^+^ and Cl^−^ ions with respect to the head group of C_10_ mim (bottom).

This binding extent of ions in the membrane was also confirmed by calculating the radial distribution functions (RDFs) of the ions with respect to the head groups of DOPC and C_10_ mim. The RDFs in Fig. 6 show that Na^+^ ions interact with the anionic phosphate groups of DOPC in the pure DOPC bilayer more strongly than in the DOPC/C_10_ mim bilayer, consistent with the results in Figs 4 and 5. Also, the RDF peak for Cl^−^ ions around the head groups of C_10_ mim is much higher than that for Na^+^ ions, showing that the interactions between C_10_ mim and Cl^−^ are much stronger than those between C_10_ mim and Na^+^; this implies that Cl^−^ ions can weaken the repulsive force between C_10_ mim and Na^+^, presumably because Cl^−^ ions, which electrostatically interact with C_10_ mim heads, can also bring Na^+^ ions to the bilayer surface. These results support the experimental results in this work, which show that the addition of C_10_ mim reduces the average conductance, because of the repulsive interactions with Na^+^ ions, and this electrostatic effect is much more significant in the fluorescence experiments (0.15 M NaCl) than in the electrical experiments (1 M NaCl). Our simulation results agree well with those of our electrical experiments, which showed reduced conductances, and fluorescence experiments, which showed decreased permeation of cations; both of these effects result in more significant interference of cation flux through a gA channels at low ionic strengths.

## Discussion

Although ILs are considered to be green solvents, ILs are toxic to aquatic environments, precluding their wide application. Toxicity analyses of ILs and the development of safer ILs are therefore needed, as a number of industrial applications of ILs have been proposed because of the advantages of ILs. In this work, we used model membranes to perform a comprehensive analysis of the effects of ILs on lipid bilayers. Our experimental results suggest that ILs incorporated into membranes influence the activities of membrane proteins by changing the physical properties of the membranes. We identified two dominant effects of ILs on membranes. First, ILs that were tested in this work decrease the membrane deformation energy, which is caused by the difference between the lengths of ion channels and lipid bilayers, resulting in prolonged channel lifetimes. Secondly, ILs create positive charges on the membrane surface, interfering with cation permeation. The ion permeation was significantly decreased when a buffer solution of low ionic strength was used. The former effect was dominant when buffer solutions with high ionic strengths were used. We further studied the observed phenomena via MD simulation. The simulation results agreed well with our experimental results. For high salt concentration buffers, the interference with cation permeation by ILs was relatively low, and decrease of the deformation energy was more effective. However, for low salt concentration buffers, interference with cation permeation by ILs was dominant. Both effects were more significant when ILs with longer alkyl chains and/or at higher concentrations were used. In summary, we show that ILs may disrupt the homeostasis of living organisms by perturbing ion channel functions, and this must be taken into account when designing greener ILs.

## Methods

### Ionic liquids

The cation alkyl chain of an IL determines the lipophilicity of the IL, i.e., the length of the alkyl chain of the IL liquid determines the hydrophobic properties of the IL. This property can be used to induce ecotoxicity by manipulating the length of the alkyl chain to optimize the bonding strength with liposomes or lipid bilayers^35^. We therefore used 1-ethyl-3-methylimidazolium (C_2_ mim) chloride (Sigma, St. Louis, MO, U.S.A), 1-butyl-3-methylimidazolium (C_4_ mim) chloride (C-TRI, Gyeonggi-do, Korea), 1-hexyl-3-methylimidazolium (C_6_ mim) chloride (Alfa Aesar, Seoul, Korea), 1-octyl-3-methylimidazolium (C_8_ mim) chloride (C-TRI, Gyeonggi-do, Korea), and 1-decyl-3-methylimidazlium (C_10_ mim) chloride (Sigma, St. Louis, MO, U.S.A). We excluded the results obtained using C_2_ mim as no significant change was observed. All ILs were dissolved at concentrations of 1 M and 0.5 M in 1 M NaCl and 10 mM HEPES, and buffered with 1 mM EDTA at pH 7.0.

### Bilayer formation and electrical measurements

For single-channel measurements, we used the painting method^36^. An aperture of size varying from 50 to 80 μm was made, using a spark generator (DAEDALON, Salem, MA, U.S.A), on 10 μm thick PTFE film (Good Fellow, Huntingdon, England). For the lipid solution, (1,2,-diphytanoyl-*sn*-glycero-3-phosphocholine (DPhPC; Avanti Polar Lipid, Inc., Alabaster, Ala, U.S.A) was dissolved in *n*-decane (MP Biomedicals, Santa Ana, CA, U.S.A) at a concentration of 30 mg/mL; gA (Sigma, St. Louis, MO, U.S.A) was dissolved in the solution at a concentration less than 10 nM. This solution was painted around the aperture of the PTFE film and was then dried for 30 min. The film was placed horizontally in the chamber, which was filled with buffer solution (1 M NaCl, 10 mM HEPES, and 1 mM EDTA, pH 7.0). The lipid solution containing 0.5 μL gA was dispensed on the aperture to form a lipid bilayer. When a gA signal was observed, depending on the frequency of the signal, different amounts of the IL were dispensed into both sides of the chamber, and the signal changes were observed. Electrical measurements were made using an Axopatch 200 b patch clamp (Molecular Devices, Sunnyvale, CA, U.S.A) and real-time optical observations were made using a microscope (Digital Blue, QX5, Marietta, GA, U.S.A). Each data point was established by averaging over 500 signals, acquired via 250 kHz sampling with a low-pass Bessel filter (1 kHz). The data were then filtered digitally from 300 to 500 Hz. Data were collected using Clampfit 10.3 (Molecular Devices, Sunnyvale, CA, U.S.A) and analyzed using the Clampex 10.3 program.

### Large unilamellar vesicles and fluorescence experiments

The dry film of 1,2-dioleoyl-sn-glycero-3- phosphocholine (DOPC; Avanti Polar Lipids, Alabaster, Ala, U.S.A) was rehydrated with 25 mM 8-aminonaphthalene-1,3,6-trisulfonate disodium salt (ANTS; Invitrogen, Grand Island, NY, U.S.A), non-membrane-permeable water-soluble fluorescent material, buffered with 100 mM NaNO_3_ and 10 mM HEPES at pH 7.0. Then, it was sequentially sonicated, frozen and thawed, and extruded to form large unilamellar vesicles (LUVs). Subsequently, LUVs was dialyzed with 140 mM NaNO_3_ and 10 mM HEPES at pH 7.0. The LUVs were incubated with gA, dissolved in 25 mM dimethyl sulfoxide (DMSO; Sigma, St. Louis, MO, U.S.A), in a darkroom for 24 h at 12 °C. The final concentration of DMSO was adjusted to be below 0.5%. The desired concentration of IL was added to the LUV solution at 20 °C over 10 min, and fluorescence changes were measured. To verify the quenching rate, LUVs containing ANTS were mixed with a mixture of 100 mM TlNO_3_, 48 mM NaNO_3_, and 10 mM HEPES (pH 7.0) buffer solution at a ratio of 5:5 and the changes in the fluorescence intensity were measured in real time using a stopped-flow spectrofluorometer (SX-20, Applied Photophysics Leatherhead, United Kingdom). Excitation was performed at 352 nm, and the fluorescence was recorded above 455 nm using a high-pass filter, with Pro-Data control software from Applied Photophysics, at a sampling rate of 5000 points/s. TlNO_3_ was purchased from Sigma and a 2–100 ms graph was fitted via the following exponential function:


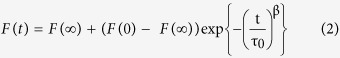


F(*t*) represents the intensity of the fluorescence at time *t*, *β* represents the dispersion of the sample data (0 < *β* ≤ 1), and *τ*_0_ represents the variable of unit time^37^. The gA activation change can be derived via the quenching rate, *k(t*), which is time dependent at 2 ms.


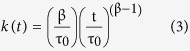


The results reported in this paper are the average (±standard deviation) of over six trials. For all ILs, concentrations from 100 μM to 50 mM were used. However, the results were excluded when the liposomes were ruptured or when a signal did not appear. Moreover, as the ILs are fluorescent, we performed normalization by subtracting the IL fluorescence data. In order to subtract IL and ANTS signals, we performed experiments without gA (–gA) for each IL. When ILs and ANTS fluorescence outside liposomes were quenched, the corresponding data were set as a base line.

### Simulations of ionic liquids with lipid bilayers

All simulations and analyses were performed using the GROMACS4.5.5 simulation package^38^,^39^,^40^. We used the OPLS all-atom force fields (FFs) for C_10_ mim and DOPC^41^,^42^, which were respectively developed by Sambasivarao and Acevedo^43^, and Tieleman *et al*.^44^ This FF previously predicted the experimentally observed areas per a lipid molecule and diffusivities of DOPC bilayers^44^,^45^. Also, the condensed-phase structures and thermodynamics of ILs and their effects on the polymer conformation agreed well with the experimental results and polymer theories^43^,^46^.

The simulated bilayer consisted of 128 DOPC molecules (or a mixture of 112 DOPC and 16 C_10_ mim) and ~5000 TIP4P water molecules in a periodic box of size 6 × 6 × 8 nm^3^. To reproduce the experimental conditions of 1 M and 0.15 M NaCl, 94 or 14 Na^+^ and Cl^−^ ions were added to the bilayer system. For the bilayer with C_10_ mim, 16 Cl^−^ counterions were added to achieve neutrality. Real-space cutoffs of 14 and 11 Å were respectively applied for Lennard-Jones (LJ) and electrostatic forces, with the inclusion of the particle mesh Ewald summation for long-range electrostatics^47^,^48^. A temperature of 298 K and a pressure of 1 bar were maintained by applying the velocity-rescale thermostat^49^ and Berendsen barostat^50^ in the NP_*xy*_P_*z*_T ensemble (semi-isotropic pressure coupling). The LINCS algorithm was used to constrain the bond lengths^51^. Simulations were performed for 40 ns with a time step of 2 fs, using computational facilities supported by the Supercomputing Center/Korea Institute of Science and Technology Information, with supercomputing resources including technical support (KSC-2014-C3-68). The final 20 ns trajectories were used for analyses.

## Additional Information

**How to cite this article**: Ryu, H. *et al*. Investigation of Ion Channel Activities of Gramicidin A in the Presence of Ionic Liquids Using Model Cell Membranes. *Sci. Rep*. **5**, 11935; doi: 10.1038/srep11935 (2015).

## Figures and Tables

**Table 1:** List of simulations.

	Name	No. of molecules in the membrane		No. of ions	
		DOPC	C_10_ mim	Na^+^	Cl^−^
DOPC membrane	DOPC-0.15	128	—	14	14
	DOPC-1	128	—	94	94
DOPC/C_10_ mim membrane	C10 m-0.15	64	64	14	30
	C10 m-1	64	64	94	110
